# Salient object changes influence overt attentional prioritization and object-based targeting in natural scenes

**DOI:** 10.1371/journal.pone.0172132

**Published:** 2017-02-21

**Authors:** Nicola C. Anderson, Mieke Donk

**Affiliations:** Department of Cognitive Psychology, VU University Amsterdam, Amsterdam, The Netherlands; University of Verona, ITALY

## Abstract

A change to an object in natural scenes attracts attention when it occurs during a fixation. However, when a change occurs during a saccade, and is masked by saccadic suppression, it typically does not capture the gaze in a bottom-up manner. In the present work, we investigated how the type and direction of salient changes to objects affect the prioritization and targeting of objects in natural scenes. We asked observers to look around a scene in preparation for a later memory test. After a period of time, an object in the scene was increased or decreased in salience either during a fixation (with a transient signal) or during a saccade (without transient signal), or it was not changed at all. Changes that were made during a fixation attracted the eyes both when the change involved an increase and a decrease in salience. However, changes that were made during a saccade only captured the eyes when the change was an increase in salience, relative to the baseline no-change condition. These results suggest that the prioritization of object changes can be influenced by the underlying salience of the changed object. In addition, object changes that occurred with a transient signal (which is itself a salient signal) resulted in more central object targeting. Taken together, our results suggest that salient signals in a natural scene are an important component in both object prioritization and targeting in natural scene viewing, insofar as they align with object locations.

## Introduction

A key question in research on attention and oculomotor control in natural scene viewing is the extent to which eye movements and attention may be governed by the low-level visual features of the scene or more high-level, cognitive factors. This has been the focus of much debate; however, it is becoming increasingly clear that while bottom-up stimulus features play a role in where people look, this is heavily influenced by cognitive factors.

Evidence that bottom-up, stimulus features influence oculomotor control stems from early attempts to model visual attention via a ‘salience map’ [1,2]. This model predicts where observers will look in an image based on differences among basic visual features such as colour, orientation and luminance. There is evidence that such models perform fairly well in predicting real fixation locations (e.g., [3–5]) and that statistical properties of an image such as higher edge density and luminance contrast correlate with fixated locations (e.g., [6,7]). In addition, when visual features such as luminance contrast and intensity are altered in selected regions of scenes, eye movement behaviour is affected [8–10].

Evidence that top-down, more cognitive factors influence oculomotor control stems from some of the earliest investigations of eye movement behavior in natural scenes [11,12], where differences in task revealed clear qualitative differences in eye movement behavior. From these early reports, more recent work has demonstrated that oculomotor control is not only influenced by task [13,14], but by a whole host of top-down factors such as context [15,16], expertise [17,18], scene semantics [19] and objects [20,21].

There is a growing number of studies demonstrating that cognitive factors play a dominant role in natural scene viewing (e.g., [22,23]) suggesting that the influence of salience on oculomotor control is marginal [7,24] or even entirely absent [25–28]. In natural scene viewing, it is generally thought that if salience is to play a role at all, usually it is only because it happens to correlate with (interesting) objects [10,20,21,26,29,30]. Strong evidence for this view derives from the finding that when fixating objects embedded within a natural scene, fixations tend to land near the center of objects; the Preferred Viewing Location (PVL) [21,29]. This is similar to the PVL typically found for words when reading [31–33], which is taken as evidence that attentional selection during reading is word-based. By extension a PVL for objects in scenes is evidence that visual selection is object, rather than feature-based. No such PVL was found for salient ‘proto-objects’ (determined by the Saliency Toolbox) unless they happened to overlap with objects [21].

One property that readily attracts attention in natural scenes is a transient signal such as a luminance change, which itself is highly salient [34,35]. For instance, Matsukura, Brockmole and Henderson [36] (see also [37–39]) presented participants with a scene in which an object could appear or change color either during a fixation (with associated transient signal) or during a saccade (without transient signal) after a pre-set amount of viewing time. Participants were asked to view the scene in preparation for a later memory test. Attentional capture was measured in terms of how much, and when, these object appearances or changes were looked at. Their results showed that both object appearances and feature changes tend to attract attention during natural scene viewing, particularly when also accompanied by a transient signal. When the transient signal was absent (the change occurred during saccadic suppression), the changed objects no longer automatically captured the gaze. Even though people were still more likely to fixate the changed location over an unchanged location, this was thought to be mediated by an identity mismatch between memory for the initial and the changed display and have little to do with low-level visual features such as salience. To more clearly investigate this, Matsukura and colleagues [36] calculated the salience of the changed objects using the Saliency Toolbox [40] and found that rates of change detection were not associated with differences in salience [36].

In contrast, it has been demonstrated in work utilizing more basic displays that changes in salience can capture the eyes even when they occur during a saccade. Silvis and Donk [41] had participants perform a visual search task for a left-tilted line in the presence of a right-tilted distractor among a background of uniform horizontal lines. Prior to search, participants had to make an eye movement from the top of the display to the bottom. The luminance of the target or distracter could be increased or decreased in such a way that during this first saccade, while masked by saccadic suppression, it became the most salient item in the display. Importantly, the target or distractor only captured the eyes if it became the uniquely most salient item in the display. This occurred even when the change was a luminance decrease, provided that it was then the most salient item in the display. The results thus showed that saccade-contingent changes capture the eyes as long as the critical change results in a uniquely high salience value. In the work of Matsukura and colleagues [36], it was unclear whether the changed object became the most salient item in the display, which could account for the discrepancy between their results and those of Silvis and Donk [41].

In the present work, we aimed to test how a change in the salience of an object embedded in a natural scene both during a fixation and during a saccade affects visual selection and object-based targeting. We asked participants to look through a scene in preparation for a later memory test. In a method similar to that of Brockmole and Henderson [37] and Matsukura and colleagues [36], we made a luminance change to an object in the scene either during a fixation or during a saccade. Crucially, the luminance change was controlled in such a way that the change was an increase in object salience or a decrease in object salience, as measured by the Saliency Toolbox [40]. This design allowed us to investigate salient changes that were associated with transient signals and those associated with salience changes per se. We investigated how these changes affected the selection of the object concerned, and how they affected object-based targeting (the PVL).

## Methods

### Participants

24 participants (ages 18–37, *M* = 21.9 years, 94% female) were recruited from VU University Amsterdam and participated in this experiment for course credit or 10.5 Euros. All reported normal or corrected to normal vision and were naive to the purpose of the experiment. The study was approved by the ethics board of the Faculty of Psychology and Education and conducted according to the principles of the Declaration of Helsinki. Participants gave written consent after being informed of the experimental procedure and at the conclusion of their participation given a verbal debriefing as to the purpose of the experiment.

### Apparatus

The experiment was designed and presented using OpenSesame [42], an open source experiment programming environment integrated with the SR Research Eyelink 1000 tracking system (SR Research Ltd., Mississauga, Ontario, Canada). Stimuli were presented on a 22-inch (diagonal) Samsung Syncmaster 2233RZ with a resolution 1,680 x 1,050 pixels and refresh rate of 120 Hz at a viewing distance of 75 cm. Eye position was recorded via a second computer at 1,000 Hz with a spatial resolution of 0.01° visual angle using a 9-point calibration and validation procedure. The eye with the best spatial accuracy as determined by the calibration procedure was chosen for tracking. The online saccade detector of the eye tracker was set to detect saccades with an amplitude of at least 0.5°, using an acceleration threshold of 9,500°/s^2^ and a velocity threshold of 35°/s. The experiments took place in a dim, sound-attenuated room. The experimenter received real-time feedback on system accuracy on a second monitor located in an adjacent room and calibration and validation were repeated as needed.

### Stimuli

Images and object annotations were selected from the LabelMe database [43]. An initial selection of the images from the database was made such that images were at least 1024 by 768 pixels in size or, if larger, conformed to a ratio of 4:3 (such that images and annotations could be resized to 1024 by 768 pixels) and that they contained at least 1 annotated object that fit inside an imaginary bounding box between 80 by 80 pixels and 250 by 250 pixels in size (in the lab, these object sizes ranged between 1.7 degrees visual angle squared to 5.4 degrees visual angle squared). Images were then converted to grayscale, reduced in luminance to 80% of the luminance value of the original image, and their corresponding salience maps were computed via the Saliency Toolbox (version 2.1) [40] using the default parameters including the features intensity and orientation weighted equally but with the color feature removed (given that the images were greyscale) and the iterative normalization type.

**Fig 1:**
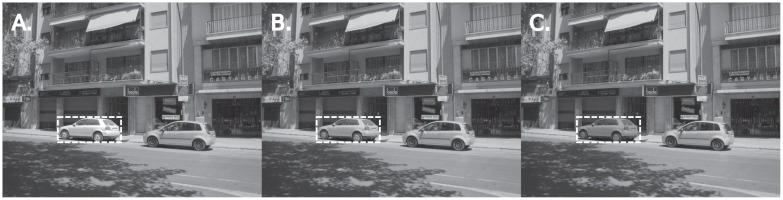
Example image with the selected object outlined in white (not shown during experiment). The critical object is increased in luminance (A), unaltered (B), and reduced in luminance (C).

Images were further selected by choosing those that contained an object that conformed to certain criteria in the following manner. An imaginary bounding box was drawn around each object (of a size between 80x80 and 250x250 pixels) and the salience value of the object was calculated from the salience map by taking the mean salience value of the area inside this box. If images contained more than one object, starting from the object with a salience value closest to the median salience of the image, the object was both increased and decreased in luminance by 25%. As the grayscale image is already reduced in luminance, this ensures that the luminance increase of the object never exceeds that of the original, unmodified image. Note that the luminance change was done on only the object itself (not to the entire bounding box area), and a Gaussian blur of approximately 10 pixels was added to the object border in order to minimize edge effects as a result of the luminance manipulation. Salience maps were re-calculated using the Saliency Toolbox for each of the resulting images with the object increased and decreased in luminance, and the mean salience value of these objects was again computed from the map. The mean salience of the luminance increased or decreased object was compared with the original salience of the unmanipulated object and if the increase in luminance resulted in an increase in salience, and a reduction in luminance resulted in a reduction in salience, their salience values were then further compared to the salience distribution of the original, unmanipulated image. The image was selected from the database if the salience of the increased luminance object was higher than the 70^th^ percentile of non-zero salience values in the original map and the salience of the corresponding reduced luminance object was below the 30^th^ percentile of non-zero salience values in the original map (1577 images were selected based on this algorithm). Some object borders outlined by the volunteers of the LabelMe project contained artefacts around the border (either including parts of background or excluding parts of the object). Therefore, images containing objects with such artefacts were excluded from the set of images, based on the first author's best judgement. This resulted in 243 images containing a critical object that conformed to these criteria. See Fig 1 for an example image.

### Procedure

Participants were seated with their head resting on a chin rest and were given verbal and written instructions regarding the experimental procedure. Calibration and validation of their eye position was performed. In the first phase of the experiment, participants were instructed to explore each of 243 images carefully so as to remember them for a later recognition task. Each trial began with a drift-correction screen in which participants were required to press the spacebar while fixating a centrally presented cross. The image containing the unaltered critical object was then presented for a randomly selected amount of time between 2000 and 4000 ms. After this time, a second image was presented either containing the luminance-altered or the unaltered critical object. There were three types of trials. In the fixation trials, the second image contained the luminance-altered object and it was presented during fixation (1/3 of trials or 81 trials). In the saccade trials, the second image also contained the luminance-altered object but it was presented during a saccade (1/3 of trials). In the remaining trials, the critical object in the second image was unaltered relative to that in the first image and provided a baseline on which the other trial types could be compared (1/3 of the trials). The timing of the second display was based on real-time feedback from the eye tracker, based on filters from the OpenSesame Eyelink toolbox, which indicted when a saccade or fixation started. Trial type was manipulated within-participants and counterbalanced across images such that each image-object pair occurred in one of these three conditions across three participants. The Direction of change (increase or decrease) was manipulated between-participants and counterbalanced across participants. For each participant, there were 243 trials in the first phase of the experiment. In the second phase of the experiment, participants performed a short memory test, where 10 images already seen by the participant were displayed (with no object change) along with 10 new images selected from unused images from the original dataset. Each image was shown for 2 seconds after which participants indicated whether they had seen the image before (press ‘z’) or not (press ‘/’). Recognition accuracy was 81.5% correct (*SE =* 2.32%). Before the experiment began, participants had a short practice session with 10 images in an encoding phase and 10 in the testing phase (5 old, 5 new). No pictures in the practice phase had an object change occur.

### Data processing

Fixations with durations shorter than 70 ms and longer than 800 ms were removed from analysis. Of the saccade trials, 74% of object changes were successfully executed during the saccade. The change was made approximately 19 ms (SD = 0.15) after the start of the saccade and average saccade duration was 64 ms. Of the fixation trials, 99% of object changes were successfully executed during the fixation. Those that were not successfully presented were excluded from analysis. To investigate how selection behavior was dynamically affected by the presence of an object change, we calculated the proportion of eye movements that landed on the critical object separately for each of the four fixations following the change occurring in the fixation and the saccade trials. For comparison, in the baseline trials, the proportions of eye movements that landed on the critical object were calculated after a randomly selected point in time between 2000 and 4000 ms (roughly equivalent to when the change occurred in the other conditions).

## Results

### Proportion of eye movements landing on object

**Fig 2:**
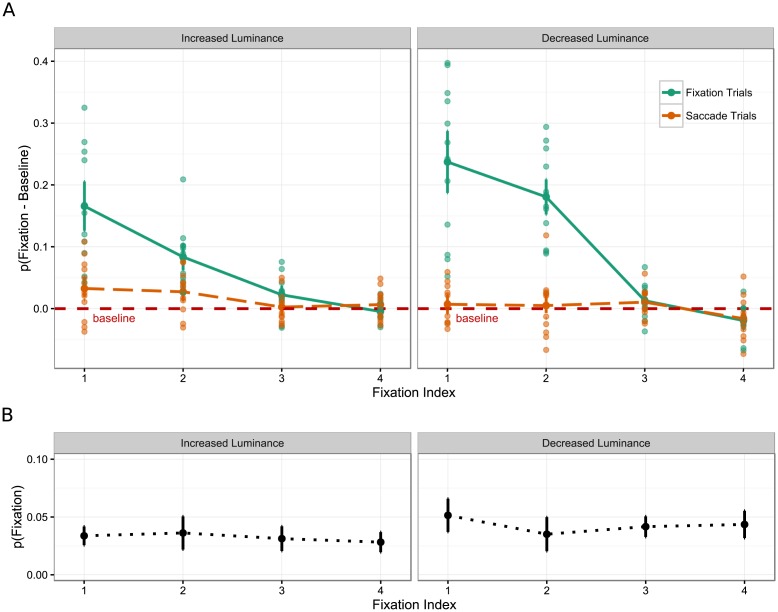
The mean proportion difference from baseline that landed within the bounding box region of the critical object. (A) For Trial type, Fixation index, and Direction of change. Individual subject mean proportion differences are plotted in lighter shaded points. (B) Baseline object viewing rates. Error bars represent confidence intervals corrected for between-subjects variance [44,45].

In order to simplify the analysis, the proportion of eye movements that landed on the critical object in the case in which no change occurred was subtracted from the proportion of eye movements that landed on the object in the case in which changes were made during a saccade and during a fixation. Thus, reported results reflect difference scores from a baseline where no change was made to the critical object. Fig 2 shows the proportion of first eye movements that landed on the critical object relative to baseline critical object fixation rates as a function of Trial type (fixation trials, saccade trials), Fixation Index (1–4), and Direction of change (increase and decrease). A 2 (Trial type) by 4 (Fixation index) by 2 (Direction of change) mixed analysis of variance was conducted on the proportion of first eye movements that landed on the object. Linear contrasts were planned for the Fixation index factor.

There was a main effect of Trial type, *F*(1, 22) = 136.178, *p* < .001, $\eta_{P}^{2}=.861$, such that the proportion of eye movements that landed on the critical object relative to baseline was higher in the fixation trials than in the saccade trials. There was a linear main effect of Fixation index, *F*(1, 22) = 72.895, *p* < .001, $\eta_{P}^{2}=.768$, such that the proportion of eye movements that landed on the critical object relative to baseline decreased linearly over fixation index. There was no main effect of Direction of change, *F*(1, 22) = 1.385, *p* = .252, $\eta_{P}^{2}=.059$.

There was no interaction between Fixation index and Direction of change, *F*(1, 22) = 2.761, *p* = .111, $\eta_{P}^{2}=.111$, however, there was an interaction between Trial type and Direction of change, *F*(1, 22) = 16.037, *p* = .001, $\eta_{P}^{2}=.422$, between Trial type and Fixation index, *F*(1, 22) = 96.299, *p* < .001, $\eta_{P}^{2}=.814$ and between Trial type, Fixation index and Direction of change, *F*(1, 22) = 8.708, *p* = .007, $\eta_{P}^{2}=.284$. To further explore this interaction, mixed analyses of variance were performed on the proportion of eye movements that landed on the critical object relative to baseline for fixation trials and saccade trials, separately. For the fixation trials, there was a main effect of Direction of change, *F*(1, 22) = 6.748, *p* = .016, $\eta_{P}^{2}=.235$, such that observers in the decrease group were more likely to select the critical object than those in the increase group. In addition, one-sample t-tests against 0 confirms that observers in both the increase group (*M* difference = 0.07), *t*(11) = 9.080, *p* < .001, and decrease group (*M* difference = 0.10), *t*(11) = 8.723, *p* < .001, were more likely than baseline to select the critical object. There was a main effect of Fixation index, *F*(1, 22) = 96.818, *p* < .001, $\eta_{P}^{2}=.815$ as well as an interaction between Fixation index and Direction of change, *F*(1, 22) = 5.660, *p* = .026, $\eta_{P}^{2}=.205$, indicating a more linear trend over Fixation index in the decrease group compared to the increase group (see Fig 2).

For saccade trials, there was also a main effect of Direction of change, *F*(1, 22) = 5.891, *p* = .024, $\eta_{P}^{2}=.211$, showing the reversed pattern in comparison to that in the fixation trials, i.e., observers in the increase group were more likely to select the critical object than those in the decrease group. In addition, one-sample t-tests against 0 confirms that observers in the increase group (*M* difference = 0.02), *t*(11) = 3.367, *p* = .006, were more likely than baseline to select the critical object, which was not the case in the decrease group (*M* difference = 0.002), *t*(11) = 0.416, *p* = .685. Moreover, there was a significant main effect of Fixation index, *F*(1, 22) = 5.479, *p* = .029, $\eta_{P}^{2}=.199$, but no interaction between Fixation index and Direction of change, *F* < 1.

In summary, object changes that were made during a fixation attracted the eyes earlier and in higher frequency than changes that occurred during a saccade (compared to baseline viewing rates). For changes that occurred during a fixation, decreases in luminance were fixated earlier and with more frequency than increases in luminance. When the object changed during a saccade, and so was masked by saccadic suppression, only increases in luminance attracted the eyes over baseline viewing rates (see Fig 2).

### Object targeting

**Fig 3:**
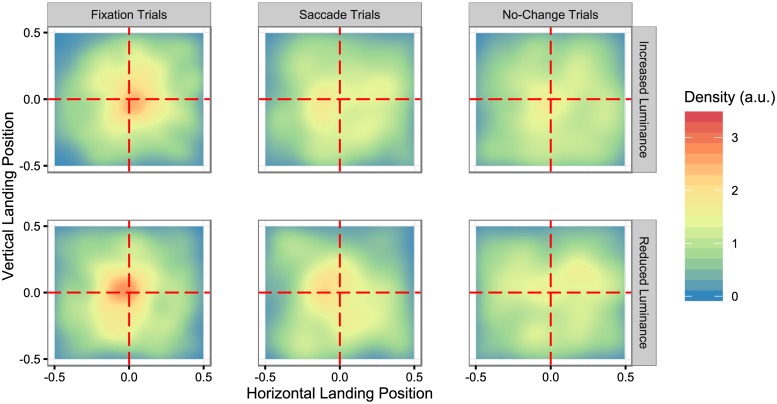
2D density plots of the normalized landing position of the first fixation into the object across Trial type and Direction of change. Density estimation for this and subsequent plots performed in ggplot2 [46] based on the R function kde2d [47].

In order to calculate normalized landing positions within objects, the first fixations that landed within the imaginary bounding box surrounding the object were selected from the original data. Normalization was conducted in a manner similar to Nuthmann and Henderson [21] and ‘t Hart, Schmidt, Roth and Einhauser [10]. The x and y pixel locations of these first fixations were recalculated with respect to a within-object coordinate system where the upper left corner of the bounding box was (0.5, 0.5) and the bottom right corner was (-0.5,-0.5). Table 1 shows the mean normalized distance from the center and Fig 3 shows the normalized landing positions of the first fixation into the imaginary bounding box surrounding the critical object after the object changed in the fixation and saccade trials or after an equivalent amount of time in the baseline trials, for the increase and decrease luminance group. Note that we collapsed across Fixation index (and included all fixations, including those beyond the 4th) to increase cell N and because there was no a-priori prediction about Fixation index on this factor (although see below for a post-hoc analysis).

**Table 1:** Mean normalized distance from object center (standard deviation in brackets) of the first fixation that landed within the imaginary bounding box surrounding the object.

Trial Type	Direction of Change	
	Increased Luminance	Decreased Luminance
Fixation	0.31 (0.02)	0.31 (0.03)
Saccade	0.35 (0.04)	0.33 (0.04)
Baseline	0.35 (0.02)	0.35 (0.03)

A mixed analysis of variance was conducted on the normalized distance from object center of the first fixations that landed within the object bounding box with the within-subjects factor Trial type (fixation, saccade, and baseline) and the between-subjects factor Direction of change (increase or decrease).

There was a main effect of Trial type, *F*(2, 44) = 13.654, *p* < .001, $\eta_{P}^{2}=.383$. There was no main effect of Direction of change, and no interaction between Trial type and Direction of change, all *F*’s < 1. Paired-samples t-tests revealed that the first fixation on the object landed closer to the center of the object for fixation trials compared to saccade trials, *t*(23) = 3.723, *p* = .001, and compared to baseline trials, *t*(23) = 5.663, *p* < .001. The first fixation did not land significantly closer to the object center for saccade trials compared to baseline trials, *t*(23) = 1.114, *p* = .277.

In summary, an object change during a fixation led to a more central within-object landing position than changes made during a saccade or when no change was made. Within-object landing position was not affected by the direction of change (see Table 1 and Fig 3).

**Fig 4:**
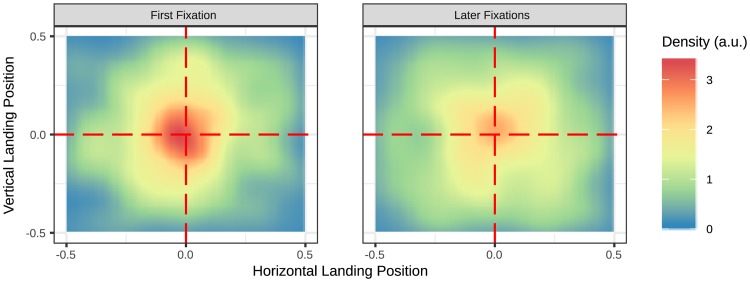
2D density plots of the normalized landing position of the first fixation into the object for fixation trials. Split into two groups: the first fixation into the object was the first fixation after the change (left) and when the first fixation that landed on the object was later on in the trial (right).

As object changes that occurred during a fixation were associated with a transient signal, the above findings led to the question of whether the salient transient itself aids in the immediate and accurate localization of the change. In order to investigate this possibility, the first fixations that landed on the object in fixation trials were further split into two groups: those fixations that immediately followed the change (i.e., when the first fixation after the change landed on the object) or those occurring later in the trial. Fig 4 shows 2D density plots for this comparison. Fixations landing on the object as an immediate response to the change (*N* = 432, *M* = 0.29), landed significantly closer to the object center than those that first landed on the object later in the trial (*N* = 690, *M* = 0.31), *t*(23) = 3.92, *p* = .001.

## Discussion

The goal of the present work was to investigate the effect of changes in object salience on both oculomotor selection and object-based targeting. We increased or decreased the salience of an object embedded in a natural scene either during a fixation (associated with a transient signal) or during a saccade (transient signal masked by saccadic suppression), and compared performance in these conditions to a baseline condition in which the critical object remained unchanged. Thus, there were two manipulations, Trial type (fixation trials, saccade trials, baseline trials) and Direction of change (an increase versus a decrease in object salience). We discuss our findings first in relation to oculomotor capture, then in relation to object-based attention/targeting.

### Oculomotor capture

Replicating previous work [36–38] Trial type affected oculomotor selection. The proportion of fixations that landed on the critical object in a scene was higher and occurred at earlier fixations when the change occurred during a fixation, than when the change occurred during a saccade, regardless of whether this change was an increase, or a decrease in salience. In addition, a change that occurred during a fixation attracted the gaze more often when the change concerned a decrease compared to an increase in salience, whereas a change that occurred during a saccade attracted the eyes more often when it concerned an increase compared to a decrease in salience.

These findings seem to be at odds with the findings of Matsukura and colleagues [36], where oculomotor capture to an object that changed during a saccade did not depend on the salience of the object. However, Matsukura et al. [36] did not manipulate the direction of the salience change resulting from their object changes. That is, they did not systematically vary the extent to which object changes led to increases or decreases in salience values. Here, we find critical differences in oculomotor capture based on both the direction of change and whether the object changed during a fixation or during a saccade. This latter finding fits with the work of Silvis and Donk [41]. In that work, a singleton that was changed during a saccade only captured the eyes when it became the most salient item in the display. These results, together with the results presented here, indicate that changes that occur without a transient signal may capture attention, but only when the change results in an item (or object) that is the most salient item in the display.

In the case where the change occurred during a fixation, attentional capture was strongest when the change was a reduction in salience, compared to an increase. At face value, this is a counterintuitive and unexpected finding. A possible explanation may be found in the magnitude of the salience change itself, calculated from the salience map. When selecting our image-object pairs, we took pains to manipulate the direction of the salience change on the basis of the output of the Salience Toolbox. Luminance modified objects were only selected such that their resulting salience map values were above the 70^th^ percentile of salience values in the map for a salience increase and below the 30% percentile of salience values in the map for a salience decrease. It may very well be the case that the magnitude of the change itself was actually higher for reductions in salience compared to increases. This fits with previous findings that different changes in object features can influence oculomotor capture [36]. However, it adds the interesting possibility that the magnitude of a salience change can influence the extent of oculomotor capture in conjunction with a transient signal (c.f., [48]).

### Object-based attention

Research to date on object-based attention in natural scenes has concluded that many (but not all) fixations in scenes target objects, and that when they do, fixations tend to land closer to the center of the objects [21,30]. This PVL for objects appears to be related to object ‘importance’ [10], is functionally related to object processing [29] and is thought to be based on the cognitive relevance of objects in scenes [22]. This latter idea implies that object identity can be determined in the periphery, an idea that is contentious in the literature (e.g., [49,50]). This has raised the speculation that some form of object processing may allow for eye guidance, without the need for full object recognition. This is thought to be possibly dependent on object salience [29], presumably where object edges act as salient reference points, with direct fixation in the middle of the object (the PVL) a result reminiscent of the global effect [51,52].

Here, we found no evidence to suggest that the direction of salience change differentially impacted the tendency for the eyes to land near the center of an object. However, we found that Trial type strongly affected the PVL. When the object changed during a fixation, the eyes landed closer to the center of the object, compared to when the change occurred during a saccade or when no change occurred. This is particularly the case when the fixation landed on the object immediately after the change, i.e., it was a direct (and first) response to the highly salient transient signal.

The former finding is in line with previous work that also statically increased the luminance of objects in scenes [10]. In that work, increasing object luminance (but not necessarily object salience) had no effect on the PVL. However, increases in object luminance tended to increase their ‘importance,’ as rated by subjects. In turn, objects higher in ‘importance’ tended to induce a more central PVL than less important objects. Although we initially hypothesized that increases in salience may affect the PVL, it is interesting to note that here, the Trial type itself had more of an impact on the PVL, rather than the direction of the salience change. One explanation of this latter finding may lie in ideas about object-based attention in work utilizing more basic displays. In such work, object-based attentional benefits tend to occur for objects that possess general feature-based properties such as having connected edges (e.g., [53,54]). Fixating the middle of an object in a natural scene (which is naturally more complex than objects used in more seminal work), would require only a rough representation of its’ shape. Perhaps here, the transient salient signal boosted the perception of such low-level shape characteristics in a manner similar to what is believed to underlie the detection of scene gist [16,29], regardless of whether the final object was increased or reduced in salience as a result.

## Conclusions

In the present work we investigated how object changes affect oculomotor capture and object targeting by increasing or decreasing an object’s salience either during a fixation (with transient signal), during a saccade (masked by saccadic suppression) or by not changing it at all. We found that, like previous work, changing an object during a fixation automatically captured the eyes. Additionally, when objects were changed during a fixation, they were more likely to capture the eyes when they decreased in luminance (and salience), a factor that may relate to the magnitude of the salience change. When an object was changed during a saccade, it only captured the eyes when the object increased in salience, suggesting a privileged status of objects that increase in salience in a display. In terms of object-based targeting, object changes during a fixation were also associated with a more central PVL, particularly when the change captured the eyes immediately. This suggests that the salient transient signal associated with such a change is a fundamental component of object targeting and object-based attention in the context of oculomotor capture. Taken together, our results suggest that salient signals do constitute a fundamental component in scene viewing insofar as salient signals are unique or transient, and correspond with object locations.

One idea emerging from work utilizing more basic displays, is that salience functions as a spatiotopic placeholder system for object localization that is consistent, and is retained over saccades [55,56], with salience influencing occulomotor capture, [41] and saccadic curvature [57,58] in subsequent saccades. In this view, effects of salience are an emergent property of the speed at which information is processed in the visual system. After some time, information concerning the relative salience of scene regions becomes information concerning their relative locations. This would explain why salience only captures attention when eye movements are initiated quickly in a new display [8,59–62] when a change is associated with a transient motion signal (e.g., [37,63]), or results in a uniquely high salience value [41]. With an attentional system largely under top-down control, salience allows for a representation that remains stable over time, but is flexible enough for the visual system to rapidly respond to new information, and quickly hand back control. In addition, this idea, and the findings of the present work, implies that salience, and low-level image features, may play an important and ongoing role in object-based attentional selection in natural scenes.
